# The Role of HER2 in Self-Renewal, Invasion, and Tumorigenicity of Gastric Cancer Stem Cells

**DOI:** 10.3389/fonc.2020.01608

**Published:** 2020-08-21

**Authors:** Li-Fei Sun, Kun Yang, Yi-Gao Wang, Yu-Xin Liu, Pei-Xian Hou, Zheng-Hao Lu, Xiao-Long Chen, Wei-Han Zhang, Zong-Guang Zhou, Xian-Ming Mo, Jian-Kun Hu

**Affiliations:** ^1^Department of Gastrointestinal Surgery and Laboratory of Gastric Cancer, State Key Laboratory of Biotherapy, West China Hospital, Collaborative Innovation Center for Biotherapy, Sichuan University, Chengdu, China; ^2^Department of Gastrointestinal Surgery, Department of General Surgery, The First Affiliated Hospital of Anhui Medical University, Hefei, China; ^3^West China School of Medicine, Sichuan University, Chengdu, China; ^4^Department of Gastrointestinal Surgery and Laboratory of Digestive Surgery, State Key Laboratory of Biotherapy, West China Hospital, Collaborative Innovation Center for Biotherapy, Sichuan University, Chengdu, China; ^5^Laboratory of Stem Cell Biology, State Key Laboratory of Biotherapy, West China Hospital, Collaborative Innovation Center for Biotherapy, Sichuan University, Chengdu, China

**Keywords:** gastric cancer, cancer stem cells, HER2, tumorigenicity, invasiveness

## Abstract

**Background:**

Deregulation of HER2 expression could affect the biological characteristics of gastric cancer cells and treatment option for gastric cancer patients. This research aims to investigate the impact of HER2 on biological characteristics of gastric cancer stem cells (GCSCs) and prognosis of gastric cancer patients.

**Methods:**

HER2 knockdown in GCSCs were constructed by lentivirus transfection. Alterations of proliferation, self-renewal, invasion, migration, colony formation, and tumorigenicity of GCSCs were examined. The changes of gene expressions after HER2 interference in GCSCs were detected by gene microarray. The impact of concentration of serum HER2 and expression of HER2 in tumor tissues on survival of 213 gastric cancer patients was also analyzed.

**Results:**

Down-regulation of HER2 decreased the self-renewal, colony formation, migration, invasion, proliferation, and chemotherapy resistance of GCSCs. However, the tumorigenicity of GCSCs *in vivo* was increased after down-regulation of HER2. The results of gene microarray showed that HER2 gene might regulate the signal transduction of mTOR, Jak-STAT, and other signal pathways and affect the biological characteristics of GCSCs. Furthermore, survival analyses indicated that patients with high concentration of HER2 in serum had a favorable overall survival. However, there was no significant correlation between expression of HER2 in tumor tissue and overall survival.

**Conclusion:**

Interference of HER2 in GCSCs decreased the capacity of self-renewal, proliferation, colony formation, chemotherapy resistance, invasion, and migration but might increase the tumorigenicity *in vivo*. Patients with high concentration of HER2 in serum seemed to have a favorable prognosis.

## Introduction

Gastric cancer (GC) is one of the most common malignant tumors in the world, with high incidence and mortality, particularly in Asian countries (1). The long-term survival rates of GC patients, especially when diagnosed with advanced disease, are still not satisfactory, although they have improved with the increase of proportion of early GC detection, the implementation of standard D2 lymphadenectomy, the development of chemotherapy, and new targeted drugs in recent years (2–4). Therefore, exploring the tumorigenic, recurrent, and metastatic mechanisms of GC is always the core of related researches and attracts great attentions. In recent years, cancer stem cells (CSCs) are considered responsible for the origin, recurrence, and metastasis of cancers because of their self-renewal, tumorigenicity, and multiple differentiation potential (5, 6). Our previous research had successfully identified and separated the gastric cancer stem cells (GCSCs) and found that GCSCs are closely involved in tumorigenesis and metastasis of GC (7). Theoretically, GCSCs are the most promising treatment candidate target for GC in the future. Investigating the alterations of signal pathways in GCSCs will be helpful to elucidate the mechanism of tumorigenesis and progress of GC and find out new effective molecular targets.

Human epidermal growth factor receptor-2 (HER2; ERBB2) plays an important role on signal transduction, proliferation, differentiation, invasion, and metastasis of cancer cells (8, 9). Studies found that overexpression of HER2 was associated with the invasion, metastasis, and poor prognosis of GC patients (10, 11). The results of the ToGA trial had further confirmed the important role of HER2 in the target therapy of GC (4).

Therefore, this research aims to investigate the impact of HER2 on proliferation, chemotherapy resistance, invasion, and tumorigenicity of GCSCs, which might shed a light on further elucidating the mechanism on how GCSCs regulate self-renewal, invasion, and tumorigenicity and show the theoretic basis of anti-tumor comprehensive therapies targeting HER2 signal pathway of GCSCs.

## Materials and Methods

### Patients and Specimens

This study collected tumor tissue samples from GC patients undergoing gastrectomy in the Department of Gastrointestinal Surgery, West China Hospital, Sichuan University, from April 2014 to December 2015. Inclusion criteria of patients were the following: diagnosis of gastric adenocarcinoma confirmed by gastric endoscopy and biopsy, and complete clinicopathological characteristics. The exclusion criteria were as follows: patients with preoperative chemotherapy or radiotherapy, patients with severe disease of other organs, and patients with any previous malignancies or synchronous malignancies. All the samples were tested by immunohistochemistry for HER2 in the Department of Pathology, West China Hospital. The blood samples of these patients were also collected before surgery in order to detect the concentration of serum HER2. Clinicopathological characteristics were retrieved from the databases and analyzed retrospectively. All the patients were followed up through outpatient service, telephone, and mail. Clinicopathologic terminology was based on the Japanese Classification of Gastric Carcinoma (3rd English version) (12). The relationships among HER2 expression level in tumor tissues, the concentration of HER2 in serum, tumor stage, and prognosis of patients were investigated.

### Cell Culture

Our previous study had identified GCSCs from tumor tissues and peripheral blood from GC patients (7). The resulting CSCs were cultured in serum-free DMEM/F12 medium (Hyclone, United States) supplemented with 20 ng/ml EGF (Peprotech, United States), 10 ng/ml b-FGF (Peprotech, United States), non-essential amino acids (Hyclone, United States), sodium pyruvate (Hyclone, United States), Glutamax (Life Technologies, United States), ITS (Sigma, United States), and B-27 supplements (Life Technologies, United States). GCSCs were cultured in ultra-low attachment dishes and incubated at 37°C in a humidified environment with 5% CO_2_. The origins of three GSCSs are described in Supplementary Table S1.

### Generation of Stable Transformants

The lentivirus target on HER2 that carried puromycin resistance gene and reporter gene was constructed by GenePharma Co. (Shanghai, China), and GCSCs were transfected with lentivirus at MOI of 20. Then, targeted cells were cultured with 5 μg/ml puromycin for 14 days or more. Efficiency of transformants was confirmed by qPCR and Western blot.

### Western Blot

The total protein of target cells was extracted by General protein extraction reagent (Bioteke Corporation, China) supplemented with protease and phosphatase inhibitor (Thermo Scientific, United States). BCA protein assay kit (Thermo Scientific, United States) was used to detect protein concentration. Proteins were loaded on 10% SDS-PAGE, transferred to 0.2-μm polyvinylidene difluoride membranes (Millipore, United States), blocked with 5% non-fat milk in TBS-T for 1 h at room temperature, and incubated with primary antibody (HER2 primary antibody: 1:1000, Cell Signaling Technology; GAPDH primary antibody: 1:5000, Sungene Biotech) at 4°C overnight and then incubated with specific secondary antibody (1:5000, Sungene Biotech). The membranes were exposed with Super Signal West Femto Maximum Sensitivity Substrate (Thermo Fisher Scientific, United States) in ChemiDoc MP Imaging System (Bio-Rad, United States).

### RT-qPCR

Total RNA was extracted by using TRI Reagent (Molecular Research Center, United States). All the instruments were RNase free. The PrimeScript RT Reagent Kit with gDNA Eraser [TAKARA Biotechnology (Dalian) Co., China] was used to degrade the genomic DNA and proceed the reverse transcription reaction and then real-time PCR reaction was conducted by using TB Green Premix Ex Taq II kit [TAKARA Biotechnology (Dalian) Co., China] through the Bio-Rad CFX Connect Real-Time PCR Detection System according to the manufacturer’s instruction. The sequences of primers were designed as follows: HER2 forward, 5′-GGCTCAGTGACCTGTTTTGG-3′, HER2 reverse, 5′-CAACCACCGCAGAGATGATG-3′; GAPDH forward, 5′-GGTGAAGGTCGGTGTGACCG-3′, GAPDH reverse, 5′-CTCGCTCCTGGAAGATGGTG-3′.

### Sphere Formation Assay

Limiting dilution assay was used to evaluate the self-renewal ability. Single-cell suspensions were diluted and seeded into a 96-well plate (100 μl per well) at a concentration of 10 cells per milliliter. Wells that contain more than one cell or those without cells were excluded. After incubation for 7–10 days, tumor spheres were observed and counted with a minimum diameter of 40 μm; sphere formation efficiency was calculated as the symbol of self-renewal ability.

### Soft Agarose Colony Formation Assay

Gastric cancer stem cells were digested to single-cell suspensions; a 300-μl suspension that contained 400 cells was mixed with equal volume of 0.7% soft agarose, and the mixture was added into a 12-well plate that was coated with 0.7% soft agarose. The plate was incubated for 2–3 weeks, and CSC medium was added every 4 days. Three replications were set for each group. The formation of colonies was observed and calculated through microscopy. Each colony should contain more than 50 cells with a minimum diameter of 40 μm for the GCSC sphere.

### Migration and Invasion Assay

The invasion assay was performed using a Transwell chamber (Corning, United States) that contained Matrigel Matrix (Corning, United States). Cells were resuspended in 100 μl of serum-free DMEM medium at a density of 3 × 10^4^ cells per well and seeded into the upper chamber that was coated with Matrigel Matrix, while the lower chamber was filled with 600 μl of DMEM medium that contained 10% FBS. After incubation for 18 h, cells on the upper surface of the membrane were removed by cotton swabs. Then, the cells on the lower surface were stained with Wright-Giemsa Stain Kit (Nanjing Jiancheng Bioengineering Institute, China), observed, and counted under a microscope. Three replications were set for each group. The migration assay was similar to the invasion assay, except that there was no Matrigel Matrix.

### CCK-8 Assay

Cell Counting Kit-8 (CCK-8, Dojindo, Japan) was used to detect the level of cell proliferation between the control group and the HER2-interfered group. GCSCs were seeded into 96-well plates (100 μl, 2000 cells per well) and five replications were set for each group. An equal volume of cell-free medium was set as a blank control group. At the indicated time point, 10 μl of CCK-8 solution was added into each well and then cells were cultured at a 37°C incubator for 120 min. The OD value was measured at 450 nm for each well.

### Chemotherapy Resistance Assay

5-fluorouracil (5-FU; Sigma, United States) and oxaliplatin (OXA; Sigma, United States) were used to evaluate the chemotherapy resistance of target cells. Cells were seeded into 96-well plates (100 μl, 2000 cells per well) and five replications were set for each groups and then cells were treated with 2.5 × 10^5^, 2.5 × 10^4^, 2500, 250, and 25 ng/ml of 5-FU and OXA for 4 days, respectively. CCK-8 assay was used to evaluate the OD value of each well.

### Xenotransplanted Tumor Models

Four-week old BALB/c nude mice were purchased from Dashuo Biotechnology Co. (Chengdu, China) and fed in a specific pathogen–free environment. HER2-interfered cells and control cells were mixed with Matrigel Matrix at a ratio of 1:1, and 100 μl of mixture that contained 10^6^ cells was subcutaneously injected to the flank regions of mice (*n* = 6 mice per group). After 3–4 weeks, the mice were sacrificed by cervical dislocation and then the tumors were removed and measured.

### Microarray Analysis

We had provided our original microarray data for further validation. It could be found on the website^1^. Changes in gene expression in HER2-interfered cells and control cells were analyzed with the GeneChip Human Transcriptome Array 2.0 (Affymetrix, United States). The microarray analysis was entrusted by Gminix (Shanghai, China).

### Statistical Analyses

The SPSS 22.0 (IBM, United States) and GraphPad Prism 5 (GraphPad Software, United States) were used to conduct the results of statistical analyses. Student’s *t* test and rank sum test were applied for continuous data analysis. The chi-square test was used for categorical data. The optimal cutoff value for serum concentration of HER2 was produced by X-tile software (version 3.6.1, Yale University). The results were treated as statistically significant only when the two-sided *p* value is less than 0.05.

## Results

### Successful Construction of Stable Transformants

**FIGURE 1:**
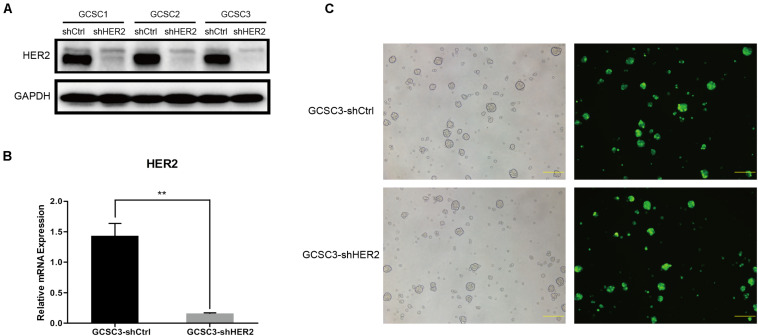
Construction of stable transformants. **(A)** Transfection efficiency detected by Western blot; GCSC3 had the highest efficiency of HER2 knockdown. **(B)** Transfection efficiency of GCSC3 detected by RT-qPCR (*p* < 0.01), **p < 0.01. **(C)** GCSC3-shCtrl and GCSC3-shHER2 under a fluorescent microscope; representative pictures were taken at ×100 magnification, and scale bars represent 200 μm.

Our study used GCSCs from three individual GC patients (GCSC1, GCSC2, and GCSC3) to detect the efficiency of HER2 knockdown by lentivirus transfection. Baseline of HER2 expression in three GCSCs is shown in Figure 1. Figure 1A shows that the protein expression level of HER2 in shHER2 was significantly lower than shCtrl in GCSC1, GCSC2, and GCSC3, while GCSC3 had the highest efficiency that would be used for subsequent experiments. The result of RT-qPCR also showed that the mRNA expression level of HER2 in GCSC3-shHER2 was significantly lower than that in GCSC3-shCtrl (*p* < 0.01, Figure 1B). Figure 1C shows that the expression of green fluorescent protein (GFP), as the product of reporter gene, was observed in GCSC3-shCtrl and GCSC3-shHER2 through a fluorescent microscope.

### Impact of HER2 on Self-Renewal, Colony Formation, Migration, and Invasion of GCSCs

**FIGURE 2:**
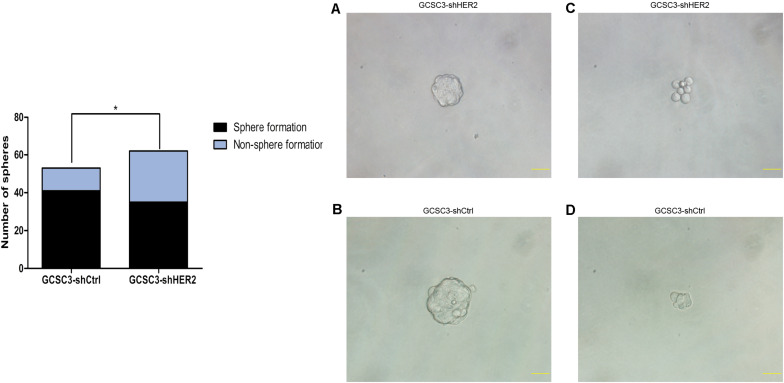
The influence of HER2 on self-renewal ability in GCSCs was examined by sphere formation assay. Representative pictures were taken at ×400 magnification, and scale bars represent 40 μm. Pictures **(A,C)** are GCSC sphere of GCSC3-shHER2 and GCSC3-shCtrl, respectively. Pictures **(B,D)** are non-sphere formation GCSC of GCSC3-shHER2 and GCSC3-shCtrl, respectively. The results showed that down-regulation of HER2 could decrease the self-renewal ability in GCSCs (77.36% vs. 56.45%, *p* = 0.029), *p < 0.05.

**FIGURE 3:**
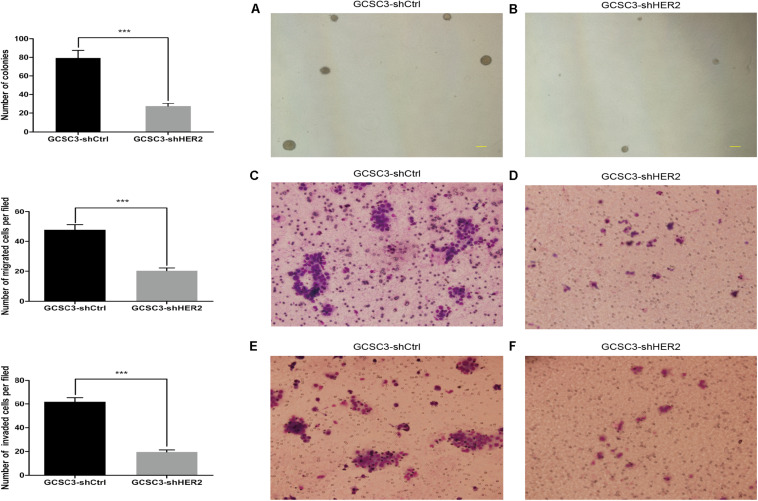
Ability of colony formation, invasion, and migration in GCSCs was detected after down-regulation of HER2. Pictures **(A,B)** show the colonies of GCSC3-shCtrl and GCSC3-shHER2, respectively. Representative pictures were taken at ×40 magnification, and scale bars represent 200 μm. The result showed GCSC3-shCtrl had a higher colony formation rate than GCSC3-shHER2 (79.33 ± 4.63 vs. 27.33 ± 1.76, *p* < 0.001); data were expressed as mean ± SEM, ***p < 0.001. Pictures **(C,D)** show that GCSC3-shCtrl had a higher capacity of migration than GCSC3-shHER2 (47.93 ± 3.38 vs. 20.27 ± 2.13, *p* < 0.001); and pictures **(E,F)** show that GCSC3-shCtrl had a higher capacity of invasion than GCSC3-shHER2 (61.87 ± 3.71 vs. 19.67 ± 1.71, *p* < 0.001). Representative pictures were taken at ×200 magnification. Data were expressed as mean ± SEM, ***p < 0.001.

By sphere formation assay, we found that the sphere formation efficiency was inhibited in GCSC3-shHER2 (35/62), compared with the control group (41/53), and the difference was statistically significant (*p* = 0.0291, Figure 2). In the soft agarose colony formation assay, the number of counted colonies was 27.33 ± 1.76 in the HER2-interfered group, compared with 79.33 ± 4.63 in the control group (*p* < 0.001, Figures 3A,B), which showed that inhibition of HER2 could decrease the ability of colony formation in GCSCs. In the migration assay, the Transwell model showed that the number of migrated cells in the control group and the HER2-interfered group was 47.93 ± 3.38 and 20.27 ± 2.13, respectively (*p* < 0.001, Figures 3C,D). For invasion assay, we found that the number of invaded cells in the control group and the HER2-interfered group was 61.87 ± 3.71 and 19.67 ± 1.71, respectively (*p* < 0.001, Figures 3E,F). Therefore, the inhibition of HER2 could significantly decrease the self-renewal, colony formation, invasion, and migration abilities of GCSCs.

### Impact of HER2 on Proliferation, Chemotherapy Sensitivity, and Tumorigenicity of GCSCs

**FIGURE 4:**
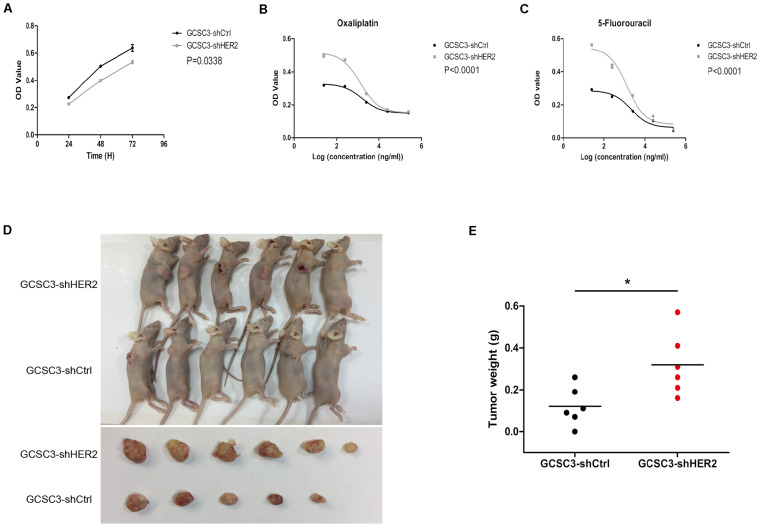
The results of CCK-8 assay, chemotherapy sensitivity, and xenograft models of GCSCs. Picture **(A)** shows that down-regulation of HER2 could decrease the proliferation of GCSCs (*p* = 0.0338). Data were expressed as mean ± SEM. Pictures **(B,C)** show that the chemotherapy sensitivity for oxaliplatin and 5-fluorouracil was increased after HER2 interference (*p* < 0.0001). Pictures **(D,E)** show that tumor weight in the GCSC3-shCtrl group was significantly lower than that in the GCSC3-shHER2 group (0.12 ± 0.04 g vs. 0.32 ± 0.06 g, *p* = 0.0192), *p < 0.05.

In the CCK-8 assay, the growth curve showed that proliferation ability of GCSC3-shHER2 decreased significantly (*p* = 0.0338, Figure 4A). According to the chemotherapy resistance assay, the results indicated that the value of IC50 for OXA in the HER2-interfered group was significantly lower than that of the control group (1282 ng/ml vs. 1609 ng/ml, *p* < 0.0001, and Figure 4B). The value of IC50 for 5-FU in the HER2-interfered group was also significantly lower than that of the control group (1323 ng/ml vs. 2087 ng/ml, *p* < 0.0001, and Figure 4C). For the model of xenograft tumor in nude mice, the tumor weight in the control group and the HER2-interfered group was 0.12 ± 0.04 *g* and 0.32 ± 0.06 *g*, respectively (*p* = 0.0192, Figures 4D,E), which demonstrated that the GCSCs have a relatively higher tumorigenicity in nude mice when the expression of HER2 was down-regulated.

### Impact of HER2 on the Prognosis of GC Patients

**FIGURE 5:**
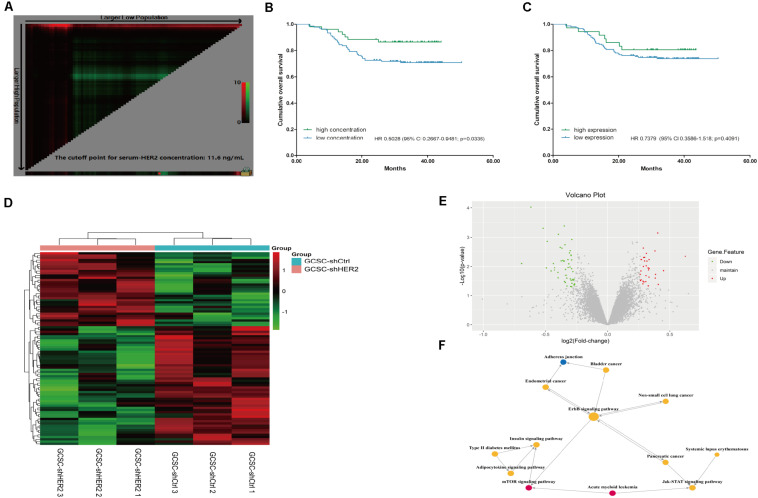
Survival analyses and results of gene microarray and signaling pathway analysis. Picture **(A)** X-tile plots for serum concentration of HER2 in our study; the red spot shows the cutoff value (11.6 ng/ml) that separates the cohort into two groups. Picture **(B)** Survival analyses of patients with different serum concentrations of HER2; the result indicated that patients with high concentration of HER2 in serum had a favorable prognosis (*p* = 0.0336). Picture **(C)** Survival analyses of patients with different expression levels of HER2 in tumor tissue (*p* = 0.4091). Picture **(D)** Heatmap representation of the gene expression profiles of HER2-interfered GCSCs and control cells. Up-regulated expressions are marked in red; down-regulations are colored green; black reflects no difference in expression levels. Picture **(E)** The volcano plot of the gene microarray is used to evaluate the overall distribution of the differential gene between the HER2-interfered GCSCs and control cells. Green plots represent down-regulation mRNA genes while red plots represent up-regulation mRNA genes with a fold change > 1.2 and *p* value < 0.05. Picture **(F)** Signaling pathway analysis revealed that HER2 gene might regulate the signal transduction of mTOR, Jak-STAT, and other signal pathways. Yellow circles represent pathways with up-regulation and down-regulation mRNA genes. Pathways that only contain up-regulation mRNA genes were marked with a red circle, while the blue one represents pathway that involves down-regulation mRNA genes. The size of circles shows the value of degree.

According to the inclusion and exclusion criteria, 213 GC patients were enrolled in the study. As of January 2018, the overall follow-up rate was 87.79% (187/213). The level of HER2 expression was detected in tumor samples and blood samples. The percentages of negative expression, HER2 1+, HER2 2+, and HER2 3+ were 42.72% (91/213), 38.50% (82/213), 16.43% (35/213), and 2.35% (5/213), respectively. For the serum concentration of HER2, the cutoff value was set as 11.6 ng/ml based on the results of X-tile software (Figure 5A), and the percentages of the high-concentration group and the low-concentration group were 29.11% (62/213) and 70.89% (151/213), respectively. We found that there was no significant correlation between the expression level of HER2 in tumor tissues and blood samples (*p* = 0.195). The high concentration of HER2 was more common in male (*p* = 0.012), and the high-concentration group had a smaller tumor size and better tumor differentiation compared with the low-concentration group (*p* = 0.021 and *p* = 0.014, respectively). This group also had a lower T stage (*p* = 0.008); however, the expression level of HER2 in tumor tissue was not correlated with tumor stage. There were only five patients with HER2 3+, so we set the subgroup that contained HER2 2+ and 3+ as HER2 high expression in tumor tissue. Clinicopathological characteristics are shown in Tables 1, 2. Furthermore, the results of survival analyses revealed that patients with a high concentration of HER2 in serum had a better prognosis compared with the low-concentration group; the hazard ratio for death in the high concentration group was 0.5028 [95% confidence interval (CI) 0.2667–0.9481; *p* = 0.0336; Figure 5B]. Nevertheless, the survival analyses showed that there was no significant difference between the different expression levels of HER2 in tumor tissues. Although the hazard ratio for death in the high expression group was 0.7379 (95% CI 0.3586–1.518; *p* = 0.4091; Figure 5C), it seemed that the prognoses of patients with 2+ and 3+ HER2 expression in tumor tissues were relatively better when compared with their counterparts.

**TABLE 1:** Clinicopathological characteristics of all patients separated by serum concentration of HER2.

Clinicopathogical features	Low concentration group ≤ 11.6 ng/mL *N* = 151	High concentration group > 1.6 ng/mL *N* = 62	*P* value
Gender (male/female)	101:50	52:10	0.012*
Age (years)	57.4 ± 11.3	57.7 ± 9.2	0.747
Tumor location (U/M/L/UML)	44:14:90:3	24:5:33:0	0.512
Tumor size (cm)	5.1 ± 2.7	4.1 ± 2.1	0.021*
Differentiation (well/poorly)	15:136	14:48	0.014*
Pathological expression level of HER2 (low/high)	126:25	47:15	0.195
T stage			0.008*
T1	23 (15.23%)	15 (24.19%)	
T2	26 (17.22%)	12 (19.35%)	
T3	42 (27.81%)	25 (40.32%)	
T4	60 (39.74%)	10 (16.13%)	
N stage			0.165
N0	44 (29.14%)	26 (41.94%)	
N1	20 (13.25%)	10 (16.13%)	
N2	29 (19.21%)	11 (17.74%)	
N3	58 (38.41%)	15 (24.19%)	
M stage			0.947
M0	131 (86.75%)	54 (87.10%)	
M1	20 (13.25%)	8 (12.90%)	
TNM stage			0.118
I	31 (20.53%)	22 (35.48%)	
II	34 (22.52%)	13 (20.97%)	
III	66 (43.71%)	19 (30.65%)	
IV	20 (13.25%)	8 (12.90%)	

*P < 0.05, statistical significance.

**TABLE 2:** Clinicopathological characteristics of all patients separated by pathological level of HER2.

Clinicopathological features	Low expression group HER2 (0/1 + ) *N* = 173	High expression group HER2 (2 + /3 + ) *N* = 40	*P* value
Gender (male/female)	122:51	31:9	0.376
Age (years)	57.1 ± 11.3	59.1 ± 8.0	0.369
Tumor location (U/M/L/UML)	47:16:107:3	21:3:16:0	0.025*
Tumor size (cm)	4.9 ± 2.7	4.4 ± 1.9	0.620
Tumor differentiation (well/poorly)	21:152	8:32	0.191
Concentration of HER2 in serum (low/high)	126:47	25:15	0.195
T stage			0.204
T1	33 (19.08%)	5 (12.50%)	
T2	31 (17.92%)	7 (17.50%)	
T3	49 (28.32%)	18 (45.00%)	
T4	60 (34.68%)	10 (25.00%)	
N stage			0.161
N0	59 (34.10%)	11 (27.50%)	
N1	21 (12.14%)	9 (22.50%)	
N2	30 (17.34%)	10 (25.00%)	
N3	63 (36.42%)	10 (25.00%)	
M stage			0.241
M0	148 (85.55%)	37 (92.50%)	
M1	25 (14.45%)	3 (7.50%)	
TNM stage			0.956
I	44 (25.43%)	9 (22.5%)	
II	37 (21.39%)	10 (25.00%)	
III	69 (39.88%)	16 (40.00%)	
IV	23 (13.29%)	5 (12.50%)	

*P < 0.05, statistical significance.

### The Variations of mRNA Expressions Detected by Gene Microarray After HER2 Interference in GCSCs

Considering the inconsistent results between *in vitro* and *in vivo* experiments, gene microarray analysis was used to verify the reason for the difference. We found that a total of 86 mRNAs were significantly differentially expressed in the two groups (fold change > 1.2; *p* < 0.05). According to the results of gene microarray analysis, the down-regulation genes in the control group contained tumor growth-related genes, protein phosphorylation-related genes, drug transmembrane transportation-related genes, and signal transduction-related genes (Figures 5D,E). Signaling pathway analysis revealed that HER2 gene might regulate the signal transduction of mTOR, Jak-STAT, and other signal pathways, and affect the biological characteristics of GCSCs (Figure 5F).

## Discussion

Nowadays, CSCs were identified and considered as one of the most important reason for tumor occurrence, development, metastasis, and recurrence. CSCs might also serve as a detection, therapy target for malignant tumor patients (13, 14). HER2 is a member of the ERBB family; activation of the HER2 can induce the self-tyrosine phosphorylation and subsequently activate several signal transduction pathways, including the Ras/MAP kinase cascade, phosphatidylinositol 3-kinase, and phospholipase C pathways, which can ultimately influence the proliferation, adhesion, differentiation, and metastasis of tumor cells (15). However, the impact of HER2 on biological characteristics of GCSCs is still unclear.

In the present study, we conducted a series of assays to investigate the role of HER2 in GCSCs. Through the CCK-8 assay, soft agarose colony formation assay, and Transwell model, we found that tumor cells with HER2 interference have a low capacity of proliferation, colony formation, invasion, and migration, which was consistent with previous studies (16, 17). We have repeated our *in vitro* researches in another primary tumor sample and found similar results (Supplementary Figure S1). Qi et al. also showed that Cullin 4B could up-regulate HER2 expression and promote invasion, clonogenicity, and proliferation in GC cells (18).

Furthermore, we found that the down-regulation of HER2 could reduce the chemotherapy resistance of GCSCs. Tomioka et al. found that inhibition of the HER2-mTOR signal might enhance fluorouracil-induced apoptosis in GC cells with HER2 amplification (19). Liu et al. demonstrated that Trastuzumab, the monoclonal antibody against HER2, increased the sensitivity of HER2-amplified human GC cells to OXA and cisplatin by affecting the expression of telomere-associated proteins (20). In ovarian cancer, overexpression of HER2 was considered to be correlated with chemotherapy resistance and stemness (21, 22).

Interestingly, the result of xenotransplanted animal tumor models showed that the interference of HER2 in GCSCs could increase the tumorigenicity *in vivo*. In addition, we found that GC patients with high concentration of HER2 in serum had a favorable overall survival. Although the survival analyses showed that there was no significant differences between the different expression levels of HER2 in tumor tissues, which is in accordance with other studies (23, 24), it seemed that the prognoses of patients with 2^+^/3^+^ HER2 expression in tumor tissues were relatively better when compared with their counterparts, which corresponded to the result of xenotransplanted animal tumor models.

Several reasons may explain the discrepancy between the results *in vivo* and *in vitro*. Firstly, the results of microarray analysis demonstrated that HER2 could function in cell growth regulation as well as protein phosphorylation; meanwhile, the mTOR signal transduction pathway may play a regulation role in it, which reveals that the different biological features between the HER2-interfered group and the control group may be associated with the HER2-mTOR signal pathway. From the literature review, we know that the HER2-mTOR signal pathway participates in the regulation of cell proliferation, tumorigenesis, invasion, and autophagy, especially for the regulation of autophagy in GC (25–27). Moreover, our unpublished data have shown that the autophagy of GCSC was associated with its tumorigenicity, rather than invasion and migration. Accordingly, the HER2-mTOR signal pathway-mediated autophagy might be one of the possible reasons why HER2 had different effects on the tumorigenicity and invasion as well as self-renewal in GCSCs. HER2 heterogeneity in GCSCs might be another possible explanation. Heterogeneous expression of HER2 within the primary tumor and between primary tumor and metastases has now been reported widely in GC (28), while the prognostic value of HER2 and HER2 heterogeneity also generated controversial results in GC (29–32). Although the mechanisms are still largely unknown, this morphologic and prognostic heterogeneity represents an intrinsic molecular complexity and heterogeneity (29). Therefore, possible explanations for discrepancies of the results *in vivo* and *in vitro* might be a consequence of intratumor heterogeneity of HER2, or genetic drift or clonal selection of HER2 during tumor progression (28).

There are also some limitations of this research. Firstly, the cases showing 2 + expression of HER2 by immunohistochemistry were not additionally examined by fluorescence *in situ* hybridization routinely because of the economic factor. Secondly, the isomers of HER2 were not considered in the study. Finally, the mechanism about how the HER2-mTOR signal pathway-mediated autophagy regulates the tumorigenicity of GCSCs will be investigated in the following researches. In this manuscript, although others have demonstrated that co-overexpression of ErbB1 and ErbB3 can be used as a prognostic factor in GC (33), we did not examine the correlation among the expression of ErbB1 and ErbB3 of gastric stem cells because some published studies have demonstrated that adding the agents targeting the ErbB1 or ErbB3 to chemotherapy does not improve overall survival or disease control rate compared with chemotherapy alone in clinical practice (34, 35), while Trastuzumab targeting the HER2 in combination with chemotherapy could improve the overall survival of patients with advanced GC (4). Therefore, we focused on investigating the role of HER2 in self-renewal, invasion, and tumorigenicity of GCSCs. Finally, the clinical significance of serum HER2 as a predictive marker for tissue HER2 and a prognostic factor should be investigated further in large sample size researches, since there were inconsistent published results. Some researches indicated that it could be a potential biomarker and used as a diagnostic marker for tissue HER2 status in GC (36, 37). However, our results has shown that there was no significant correlation between the expression level of HER2 in tumor tissues and blood samples. Also, some researches found that serum HER2 cannot be substituted for tissue HER2 or only demonstrated moderate diagnostic performance in GC (38, 39). Regarding the survival analyses, our results revealed that patients with high concentration of HER2 in serum had a better prognosis compared with the low-concentration group. However, Shi et al. reported that high serum HER2 had a negative impact on overall survival of the patients (37). Therefore, the clinical application of serum HER2 is yet to be warranted.

## Conclusion

In conclusion, our study demonstrated that down-regulation of HER2 in GCSCs could inhibit the proliferation, colony formation, self-renewal, migration, and invasion of GCSCs and chemotherapy resistance. However, the tumorigenicity of GCSCs *in vivo* was increased. GC patients with high concentration of HER2 in serum might have a favorable prognosis.

## Data Availability Statement

The original contributions presented in the study are included in the article/Supplementary Material, further inquiries can be directed to the corresponding author/s.

## Ethics Statement

This study was approved by The West China Hospital research ethics committee. Written informed consent was waived per the committee approval because of the retrospective nature of the analysis.

## Author Contributions

J-KH, Z-GZ, X-MM, L-FS, KY, and Y-GW made substantial contributions to conception and design of this study. L-FS, KY, and Y-GW conducted all the experiments. X-LC, W-HZ, Y-XL, and P-XH acquired and analyzed data. L-FS and Y-GW drafted the article. L-FS, KY, Y-GW, J-KH, and X-MM gave critical revision for important intellectual content. W-HZ, X-LC, Z-HL, Z-GZ, and J-KH critically revised the manuscript for important intellectual content. J-KH, X-MM, and KY gave the final approval of the version to be published. All authors contributed to the article and approved the submitted version.

## Conflict of Interest

The authors declare that the research was conducted in the absence of any commercial or financial relationships that could be construed as a potential conflict of interest.

## Supplementary Material

FIGURE S1 Ability of colony formation, invasion and migration in another GCSCs were detected after down-regulation of HER2. Picture **(a,b)** showed the colonies of GCSC1-shCtrl and GCSC1-shHER2, respectively. Representative pictures were taken at ×40 magnification. The result showed GCSC1-shCtrl had a higher colony formation rate than GCSC1-shHER2 (*p* < 0.005). Picture **(c,d)** showed that GCSC1-shCtrl had a higher capacity of migration than GCSC1-shHER2 (*p* < 0.005); picture **(e,f)** showed that GCSC1-shCtrl had a higher capacity of invasion than GCSC1-shHER2 (*p* < 0.005). Representative pictures were taken at ×200 magnification. In addition, the analyses of CCK-8 assay and chemotherapy sensitivity were also re-performed in GCSC1, and the similar results to the GCSC3 was found.

TABLE S1 Case characteristics of three gastric cancer stem cells.

## Abbreviations

5-FU: 5-fluorouracil
CSCs: cancer stem cells
GC: gastric cancer
GCSCs: gastric cancer stem cells
HER2: Human epidermal growth factor receptor-2
OXA: oxaliplatin.
