# Supplementary figures and images for: The Role of HER2 in Self-Renewal, Invasion, and Tumorigenicity of Gastric Cancer Stem Cells
Source: Front Oncol. 2020 Aug 21;10:1608. doi: 10.3389/fonc.2020.01608 (PMC7472958; doi:10.3389/fonc.2020.01608)

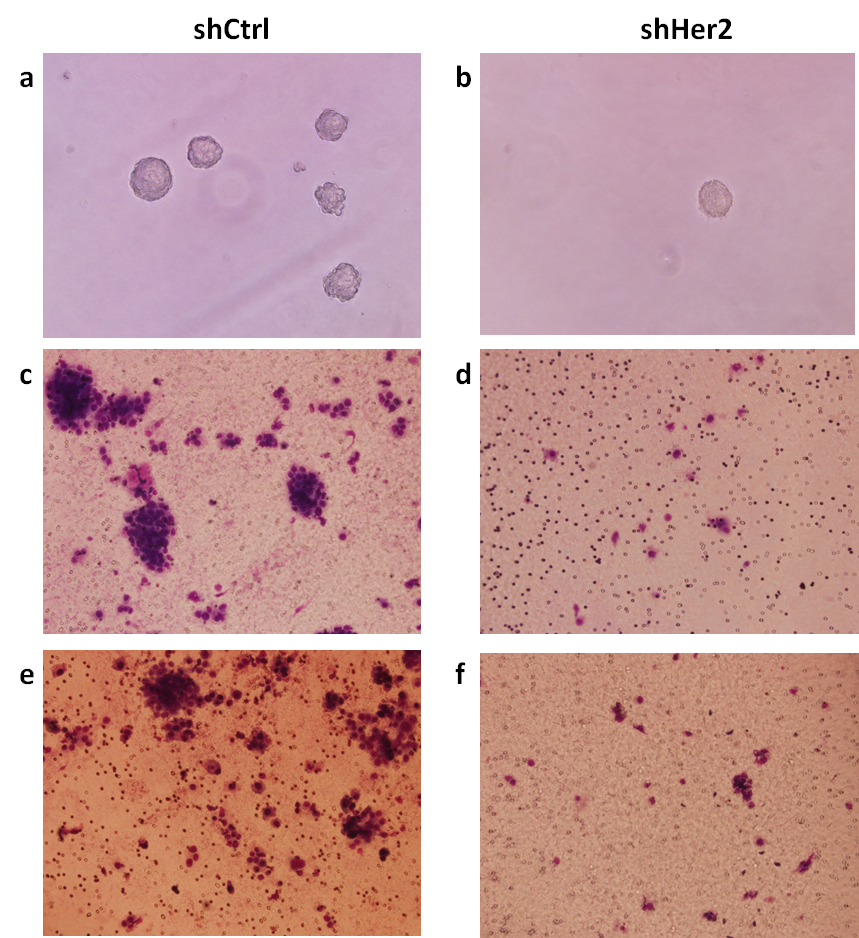

Supplement: FIGURE S1 — Ability of colony formation, invasion and migration in another GCSCs were detected after down-regulation of HER2. Picture (a,b) showed the colonies of GCSC1-shCtrl and GCSC1-shHER2, respectively. Representative pictures were taken at ×40 magnification. The result showed GCSC1-shCtrl had a higher colony formation rate than GCSC1-shHER2 (p < 0.005). Picture (c,d) showed that GCSC1-shCtrl had a higher capacity of migration than GCSC1-shHER2 (p < 0.005); picture (e,f) showed that GCSC1-shCtrl had a higher capacity of invasion than GCSC1-shHER2 (p < 0.005). Representative pictures were taken at ×200 magnification. In addition, the analyses of CCK-8 assay and chemotherapy sensitivity were also re-performed in GCSC1, and the similar results to the GCSC3 was found. [file Image_1.tif]
