# Supplementary material for: The Role of HER2 in Self-Renewal, Invasion, and Tumorigenicity of Gastric Cancer Stem Cells
Source: Front Oncol. 2020 Aug 21;10:1608. doi: 10.3389/fonc.2020.01608 (PMC7472958; doi:10.3389/fonc.2020.01608)
Supplement: TABLE S1 — Case characteristics of three gastric cancer stem cells. [file Table_1.docx]

Table S1. Case characteristics of three gastric cancer stem cells

| Case | GCSCs | Sex | Age | Grade | Tumor Location | 7^th^ AJCC Stage |
| --- | --- | --- | --- | --- | --- | --- |
| 1 | GCSC1-0603 | Male | 60 | Moderate/Poor | cardia | T4aN2M0 |
| 2 | GCSC2-112 | Male | 59 | Moderate | cardia | T1bN0M0 |
| 3 | GCSC3-0622 | Male | 51 | Moderate/Poor | cardia | T4bN1M0 |
